# The Interrelationship Between Emotional Intelligence, Self-Efficacy, and Burnout Among Foreign Language Teachers: A Meta-Analytic Review

**DOI:** 10.3389/fpsyg.2022.913638

**Published:** 2022-06-28

**Authors:** Yabing Wang, Yangyu Wang

**Affiliations:** ^1^Center for Linguistics and Applied Linguistics, Guangdong University of Foreign Studies, Guangzhou, China; ^2^School of English Education, Guangdong University of Foreign Studies, Guangzhou, China; ^3^Higher Education Mega Center, Guangdong University of Foreign Studies, Guangzhou, China; ^4^School of Foreign Languages, South China University of Technology, Guangzhou, China

**Keywords:** self-efficacy, emotional intelligence, burnout, foreign language teachers, meta-analysis

## Abstract

The importance of teachers’ affective, cognitive, and motivational factors in students’ academic achievement and well-being has been widely acknowledged. These factors are of great relevance, especially in the foreign language learning context wherein interaction between teachers and students is frequent and varies in forms. Though abundant evidence points to the high prevalence and risky factors of burnout among such a group, the extant literature lacks a quantitative synthesis of the interrelationship between emotional intelligence (EI), self-efficacy (SE), and burnout among them. This study aims to fill this research gap. The current meta-analysis is based on 42 independent samples with the experiences of 5,665 teachers reviewed. Results demonstrated moderate to large meta-correlations between these three constructs in the expected directions. EI and SE are positively correlated with each other, but negatively correlated with burnout. Moderation analysis also provides exploratory insights into the effects. Implications and future directions are also discussed.

## Introduction

There is abundant evidence documenting the importance of teachers’ affective, cognitive, and motivational factors in students’ academic outcomes (e.g., Roorda et al., 2011) and teachers’ well-being (Khani and Mirzaee, 2015). Teaching is not confined to the transmission of knowledge. In addition to teachers’ pedagogical content knowledge and skill, effective teaching constitutes teachers maintaining and transmitting positive psychological states or even dispositions, such as confidence, motivation, and enthusiasm (Moafian and Ghanizadeh, 2009). The case is especially applicable in the context of foreign language learning, wherein teacher-student communication is more frequent, divergent, and in-depth for the sake of sufficient language input and output.

However, stress and mental health among language teachers are alarming, with a large number of them reporting a wide range of psychological symptoms including frustration, anxiety, depression, and burnout (Vaezi and Fallah, 2011; Khani and Mirzaee, 2015). Among them, burnout has been increasingly studied in the field of second language acquisition because of its high prevalence and pervasive harm. As a global concern, teacher burnout has been linked to a range of adverse outcomes across every stage of teachers’ careers (Aloe et al., 2014). For example, teachers with burnout often report co-morbid psychological symptoms (Wang et al., 2021), decreased job satisfaction (Skaalvik and Skaalvik, 2009), and turnover intention (Russell et al., 2020).

In the field of second language acquisition, although a bulk of research is aimed at examining students’ emotional well-being, studies investigating teachers’ burnout have also accrued due to the positive psychology movement. Many studies were conducted aiming to answer the question of why language teachers do not react uniformly to work stressors and who are more susceptible to the development of burnout. Though abundant evidence suggested that, in addition to workplace factors (such as workload and student misbehavior), personal self-beliefs and dispositions could serve as antecedents of teacher burnout; few studies have systematically synthesized psychological antecedents of language teachers’ burnout. This study aimed to fill this research gap by meta-analyzing available empirical studies linking self-efficacy, emotional intelligence, and burnout among foreign language teachers.

## Literature Review

### Teacher Burnout

The concept of job or occupational burnout was developed and popularized by Freudenberguer (1974) and Maslach (1976) when they observed fatigue or frustration due to professional factors. It is, although defined inconsistently, composed of tripartite symptom clusters: emotional exhaustion, depersonalization, and reduced personal accomplishment (Maslach et al., 1996a), with the first two components as the central ones according to some researchers (Schaufeli and Salanova, 2007). As for teachers, emotional exhaustion refers to a state of emotional fatigue due to factors such as educational overload. It is characterized by feelings of listlessness and low enthusiasm. Depersonalization refers to a distant, indifferent, and negative attitude toward students and school. Reduced personal accomplishment denotes self-assessment of low (or lowered) teaching competence. It is, as a correlate of the first two dimensions, replete with a sense of self-doubt, and inefficiency.

Burnout has been reported among many human service professionals worldwide including doctors, firefighters, and teachers. Though reasons may vary, all teachers experience some degree of occupational stress (Jennett et al., 2003). Such stress could be successfully copied by most teachers but can burn out those who could not. Evidence showed that, in comparison with other fields, teachers tended to score higher in emotional exhaustion, and depersonalization and lower in personal accomplishment (Maslach et al., 1996a). As a result, the teacher attrition rate was high and exhibited an upward tendency over the first three working years (Boyd et al., 2008).

Given its importance, burnout has become an important research topic in academic settings. It has been attributed to both organizational (e.g., workload, school climate, student misbehavior) and individual factors (e.g., age, gender, personality, self-efficacy) (Mérida-López and Extremera, 2017). In the field of foreign language learning, studies investigating its antecedents have also proliferated, especially for two personal resource variables: emotional intelligence and self-efficacy (Ju et al., 2015; Khani and Mirzaee, 2015; Amirian et al., 2021). Although two review articles are pointing to the negative associations between emotional intelligence and burnout (Mérida-López and Extremera, 2017) and classroom management self-efficacy and burnout (Aloe et al., 2014), no study has ever meta-analyzed the interrelationship between these three variables among foreign language teachers.

### Emotional Intelligence and Burnout

Emotional intelligence (EI) has been referred to as the extent to which individuals could deal with affective information, such as emotion identification, understanding, monitoring, and regulation (Mayer et al., 2016). Conceivably, individuals with high EI are better able to get their own and others’ emotional information and use it to guide their thinking and behaviors. Extant literature has distinguished it into a trait (or mixed) and ability EI (MacCann et al., 2020). Based on the ability model, EI refers to a set of abilities related to emotional information processing (Mayer et al., 2016). Trait EI, however, is viewed as a lower-order stable personality trait encompassing a mix of emotion-related dispositions (Bar-On, 2000).

The consideration of teachers’ EI was important, even though neglected because emotionally intelligent teachers are more likely to manage emotions in the classroom and provide students help in this regard (Rastegar and Memarpour, 2009). There is abundant evidence that EI was negatively associated with stress and burnout among teachers instructing foreign languages (Vaezi and Fallah, 2011; Amirian et al., 2021) and other subjects (Skaalvik and Skaalvik, 2009; Mérida-López and Extremera, 2017). At the same time, EI training has been considered helpful to enhance teachers’ stress resilience and well-being (Vesely et al., 2014).

In the field of foreign language instruction, researchers have underlined the importance of understanding language teachers’ emotions (Schutz and Lee, 2014) since “language teaching is a highly emotional job” (Cowie, 2011). Even though the contribution of EI to language teachers’ burnout has been consistently supported in empirical studies (Vaezi and Fallah, 2011; Heiran and Navidinia, 2015), we know little about the potential moderators of their relationship, which poses a big challenge for future research.

### Self-Efficacy and Burnout

Grounded in the social cognitive theory (Bandura et al., 1999), self-efficacy is conceptualized as individuals’ judgment of their capabilities to organize and execute courses of action to attain the desired achievement. In educational settings, teachers’ self-efficacy is a multidimensional construct, referring to their self-evaluation of capabilities to accomplish instructional tasks and achieve desired outcomes of student and classroom management (Tschannen-Moran et al., 1998). It has been related to a range of teacher outcomes, such as persistence, enthusiasm, and commitment, as well as student outcomes including motivation, academic achievement, and self-efficacy (Tschannen-Moran and Hoy, 2001; Jerald, 2007).

Literature has a well-documented negative relationship between teacher’s self-efficacy and burnout across different educational stages, subjects, and countries (Schaufeli and Salanova, 2007; Skaalvik and Skaalvik, 2007; Malinen and Savolainen, 2016; Zhu et al., 2018). Many researchers strived to explain this relation. For example, Bandura et al. (1999) believed that less self-efficacious teachers tended to magnify and overreact to environmental stress, threats, and dangers. Brouwers and Tomic (2000) attributed it to teachers’ blame on students, such that teachers with low self-efficacy were more likely to blame students for management deficiency and, therefore, develop negative attitudes toward students and feel emotionally exhausted.

In the field of foreign language teaching, teachers are, even though systematically trained, usually non-native speakers of the language taught, and, thus, exposed to additional threats to self-efficacy and burnout, such as limited exposure to the target language and sense of language insufficiency. When teachers experience less confidence in delivering the lecture in the target language and need to monitor grammatical rules and accurate wording, they are more likely to feel emotionally drained, disengaged, and underqualified. Even though the link between self-efficacy and burnout has been reviewed among teachers in general (Brown, 2012) and established among foreign language teachers (e.g., Mashhady et al., 2012; Khani and Mirzaee, 2015), we still know little about the overall averaged magnitude of this link nor potential moderators, which calls for a quantitative review to fill this gap.

### Emotional Intelligence and Self-Efficacy

Language teachers’ empathy, emotional support, and efficacy to carry out student-student or teacher-student interaction activities are of great significance. In the extant literature, teachers’ EI and efficacy mingled and interacted with each other such that teachers who have higher control of emotions tend to develop stronger efficacy (Koçoğlu, 2011) and better relationships with students (Moafian and Ghanizadeh, 2009). The positive relation between teachers’ EI (especially the intrapersonal dimension of EI) and self-efficacy has been found across a range of samples including pre-service and in-service teachers (Chan, 2008), math, physics, and language teachers (Rastegar and Memarpour, 2009; Mouton et al., 2013; Alrajhi et al., 2017).

The link between these two positive psychological constructs is more evident in foreign language learning settings with group discussions and cooperation as the norm (Moafian and Ghanizadeh, 2009). Following the humanistic approach to education, an increasing number of studies began to investigate the relationship between language teachers’ EI and self-efficacy. Their relation is hardly surprising because teachers who could more appropriately understand their own and students’ emotions and swiftly shift negative emotions into productive ones would evaluate classroom instruction as less challenging. However, we still know little about how much variance of SE could be explained by EI among forging language teachers.

Taken together, with great relevance to language learners’ learning effect and teachers’ psychological well-being, language teacher burnout is worthy of attention. Though the importance and contribution of work-related factors have been extensively acknowledged and investigated, whether and to what extent language teachers’ strengths protect against burnout is still underexplored. Given that the extant literature provides sufficient data for a comprehensive quantitative review of how two personal factors (i.e., SE and EI) were associated with burnout, as well as how the two factors *per se* were intercorrelated, a meta-analysis in this regard is needed.

## The Current Study

This study has two aims. Firstly, concurrent interrelationships between EI, self-efficacy, and burnout among foreign language teachers were estimated based on a large study number and heterogeneous study characteristics. Secondly, between-study moderators (e.g., school type) were tested, which would provide a quantitative explanation of variation in the intercorrelation between these three variables.

## Method

### Selection of Studies

We applied the following strategies in the literature search: (a) databases PsycInfo, ERIC, EBSCO, ProQuest, and Google Scholar; (b) references in relevant review articles; (c) references of all articles included in this meta-analysis; (d) hand search for the key journals: *System; The Modern Language Journal; Applied Linguistics; Journal of Teacher Education; TESOL Quarterly; Language Teaching*, and *Teaching and Teacher Education.* For EI, we used the search string “emotional intelligence* OR emotional quotient OR EI OR emotion perception OR emotion management OR emotion recognition OR emotion regulation.” The asterisk (truncation) allowed for variations of word endings (e.g., intelligent* yielded articles with intelligent and intelligence). For burnout, we used the search string “burnout OR emotional exhaustion OR depersonalization OR personal accomplishment.” For self-efficacy, we used the search string “teacher efficacy OR teacher self-efficacy.” In addition, we confined the participatory setting to be “EFL OR L2 OR foreign language OR second language.” The search resulted in 658 citations. After removing duplicates, we reviewed the title, abstract, and full text (when necessary) of the remaining citations.

### Inclusion and Exclusion Criteria

Citations would be included in the current meta-analysis if they met the following inclusion criteria: (a) at least two of the three constructs (EI, burnout, and self-efficacy) were measured explicitly. EI could be of any type, ability, or trait, but it had to be self-reported. Student-reported data were excluded. Self-efficacy could also be in any form, be it class-management efficacy or language speaking/teaching efficacy. Studies reporting relevant but distinct constructs (e.g., spiritual intelligence) were also excluded; (b) the study was quantitative, with a zero-order correlation coefficient and enough information to calculate effect sizes; (c) results presented in tables and texts were consistent rather than contradictory (Wang et al., 2021). For example, if the paper reported a negative correlation between EI and self-efficacy in the main text, but tables suggested a reverse direction, it would be excluded; (d) the study was written in English; and (e) participants were second/foreign language teachers. In my previous reviews (Chesnut and Burley, 2015), we included both pre-service and in-service teachers. If more than one study was based on the same group of teachers, only the study with the most comprehensive information was included for the sake of effect-size independence.

### Coding of Studies

To capture potential sources of variations in effect size data, several variables were coded as moderators. Among them, categorical moderators included: (1) publication status (published vs. unpublished); (2) school type (public vs. private); (3) teacher type (pre-service vs. in-service); (4) educational stage (high school vs. language institutes vs. university); (5) EI stream (ability vs. trait EI). The distinction of the EI stream was based on extant meta-analyses on EI (Perera and DiGiacomo, 2013; MacCann et al., 2020). Continuous moderators included the following: (1) publication year; and (2) gender (proportion of female participants; range: 0–1). We recorded the directions of correlations between SE, EI, and a reduced sense of personal accomplishment (one subscale of burnout) when studies did not reverse them such that higher levels of EI and SE corresponded to higher levels of personal accomplishment.

We did not code country and examine the cultural influence as potential moderators of the correlations because there was not much variation with more than half of the studies included in this meta-analysis conducted in Iran, followed by Turkey (see Table 1 for the country origin of studies included). Similarly, age and years of teaching experience were not coded due to a lack of mean scores in most studies. Though measures of constructs (e.g., self-report vs. other-report) were frequently examined in other meta-analyses, such information was also not codable in the current study since all of the data were self-reported.

**TABLE 1:** Summary of studies included in the meta-analysis.

Studies (EI-Burnout; *k* = 9)	N	Country	Female %	School	Stage	EI stream	EI measure	Burnout measure	Correlation
Akbari and Tavassoli (2011)	264	Iran	0.78	Private	Institute	Trait	SEIS	MBI	−0.09
Alavinia and Ahmadzadeh (2012)	75	Iran	0.51	Public	High school	Trait	EQ-i	MBI-ES	−0.69*
Amirian et al. (2021)	124	Iran	0.35	Public	High school	Ability	WLEIS	MBI-ES	−0.33*
Durhan (2019)	166	Turkey	0.84	Public	High school	Ability	AES	TBS	−0.25*
Esmaili et al. (2018)	63	Iran	0.52	Private	Institute	Trait	EQ-i	MBI	−0.58*
Heiran and Navidinia (2015) (public)	61	Iran	0.65	Public	–	Trait	REIS	MBI-ES	−0.60*
Heiran and Navidinia (2015) (private)	39	Iran	0.65	Private	–	Trait	REIS	MBI-ES	−0.43*
Mahmoodi and Ghaslani (2014)	125	Iran	–	Private	Institute	Trait	EQ-i	MBI-ES	−0.36*
Vaezi and Fallah (2011)	104	Iran	0.50	Private	Institute	Trait	EQ-i	MBI-ES	−0.64*

**Studies (Self-efficacy-Burnout; *k* = 18)**	**N**	**Country**	**Female %**	**School**	**Stage**	**EI stream**	**SE measure**	**Burnout measure**	**Correlation**

Akbari and Tavassoli (2011)	264	Iran	0.78	Private	Institute	–	–	MBI	−0.29*
Akhavanattar and Ahmadi (2017)	104	Iran	–	Private	Institute	–	TSES	MBI-ES	−0.37*
Amirian et al. (2021)	124	Iran	0.35	Public	High school	–	TSES	MBI-ES	−0.29*
Fathi (2020)	213	Iran	0.57	–	–	–	TSES	MBI-ES	−0.49*
Ghasemzadeh et al. (2019)	171	Iran	0.57	Mixed	Institute	–	TSES	Adapted	−0.62*
Ghorbanzadeh and Rezaie (2016) (female)	83	Iran	1	Public	High school	–	TES	MBI	0.55*
Ghorbanzadeh and Rezaie (2016) (male)	31	Iran	0	Public	High school	–	TSES	MBI	0.77*
Gigasari and Hassaskhah (2017)	279	Iran	0.75	Mixed	Mixed	–	ITSES	MBI	−0.29*
Khani and Mirzaee (2014)	216	Iran	–	Private	Institute	–	TSES	MBI	−0.50*
Mardani et al. (2015)	55	Iran	0.51	Public	High school	–	TEBS	MBI	−0.36*
Mashhady et al. (2012)	112	Iran	0.50	Private	Institute	–	TSES	MBI-ES	−0.61*
Mede (2009)	63	Turkey	0.56	Public	University	–	TISES	MBI-ES	−0.12
Motallebzadeh et al. (2014)	200	Iran	0.76	–	–	–	TSES	MBI-ES	−0.58*
Ozkara (2019)	118	Turkey	0.73	Mixed	Mixed	–	JULTEB	MBI	−0.27*
Roohani and Iravani (2020)	80	Iran	0.45	Public	High school	–	TSES	MBI-ES	−0.67*
Safari (2021)	152	Iran	0.41	Mixed	Mixed	–	TSES	MBI-ES	−0.48*
Topuzov et al. (2020)	205	Ukraine	0.93	Mixed	Mixed	–	GSES	MBI	−0.18*
Yazdi et al. (2014)	616	Iran	0.68	–	–	–	Self-made	MBI	−0.61*

**Studies (Self-efficacy-EI; *k* = 15)**	**N**	**Country**	**Female %**	**School**	**Setting**	**EI stream**	**Measure of SE**	**Measure of EI**	**Correlation**

Akbari and Tavassoli (2011)	264	Iran	0.78	Private	Institute	Trait	–	SEIS	0.21*
Amirian et al. (2021)	124	Iran	0.35	Public	High school	Ability	TSES	WLEIS	0.63*
Amirian and Behshad (2016)	70	Iran	0.63	Public	Mixed	Ability	TSES	AES	0.67*
Karakas (2016)	207	Turkey	0.68	Public	University	Trait	TSES	EQ-i	0.42*
Koçoğlu (2011)	90	Turkey	0.88	Public	University	Trait	TSES	EQ-i	0.31*
Kostić-Bobanović (2020)	213	Croatia	0.71	Public	Mixed	Trait	TSES	TEIQue	0.37*
Mashhady (2013)	71	Iran	0.37	Private	Institute	Trait	TSES	EQ-i	0.71*
Moafian and Ghanizadeh (2009)	89	Iran	0.82	–	–	Trait	TSES	EQ-i	0.53*
Nikoopour et al. (2012)	336	Iran	0.68	Private	Institute	Trait	TSES	TEIQue	0.54*
Özel (2019)	200	Turkey	0.70	Public	University	Trait	TSES	SSEIT	0.62*
Rastegar and Memarpour (2009)	72	Iran	0.50	Public	High school	Trait	TSES	EIS	0.50*
Moghadam (2015)	100	Iran	0.50	Public	High school	Trait	TSES	EQ-i	0.79*
Sarkhosh and Rezaee (2013)	105	Iran	–	Public	University	Trait	TSES	EQ-i	0.53*
Siyamaknia et al. (2013)	102	Iran	–	Public	Mixed	Trait	TSES	EQ-i	0.69*
Wossenie (2014)	43	Ethiopia	–	Public	Primary	–	TSES	EIQ	0.15*

SEIS, Schutte Emotional Intelligence Scale; WLEIS, Wong and Law Emotional Intelligence Scale; EQ-i, Emotional Quotient Inventory; AES, Assessing Emotions Scale; REIS, Revised Emotional Intelligence Scale; TSES, Teacher Self-Efficacy Scale; JULTEB, Japanese University Language Teacher Efficacy Beliefs; GSES, General Self-efficacy Scale; TES, Teacher Efficacy Scale; TISES, Teacher Interpersonal Self-efficacy Scale; ITSES, Instructional Self-efficacy Scale; TEIque, Trait Emotional Intelligence Questionnaire; SSEIT, Schutte Self-report Emotional Intelligence Test; EIS, Emotional Intelligence Scale; EIQ, Emotional Intelligence Questionnaire; MBI, Maslach Burnout Inventory; MBI-ES, Maslach Burnout Inventory-Educators’ Survey. * Indicates significant results.

### Data Analysis

This study followed the method prescribed by Hunter and Schmidt (2004). The random-effect model was applied since it accounted for not only sampling errors, but also between-study variance and was believed to be stricter than the fixed effect model (Raudenbush, 2009). Pearson correlation (r) was chosen as the effect size statistic. To address the skewed distribution, *r* coefficients were transformed to *Z via* Fishers’ *Z* transformation with corresponding inverse variance weights (ω = n−3) and back-transformed to *r* when reporting effect size point estimates (Lipsey and Wilson, 2001). All of the analyses were done on SPSS (v.25) and Comprehensive Meta-Analysis (CMA2.0).

In addition to weighted mean effect sizes, heterogeneity of studies was also examined. When there was a high degree of heterogeneity between studies (*I*^2^ ≥ 75%; *Q* significant), moderator analysis would be conducted to capture the potential source of it. *Q* statistic was supplemented with *I*^2^, which could indicate the magnitude of the heterogeneous dispersion (Lipsey and Wilson, 2001). Meta-regression analysis was ran when the moderator was continuous (e.g., age) and sub-group analysis was ran when the moderator was categorical (e.g., publication status).

Publication bias was tested to see if there was an overestimation of effect sizes with significant results more likely to be published. Both published and unpublished studies (e.g., master’s and doctoral theses) were searched and included in this study as a strategy to address potential publication bias. We further examined it with a funnel plot, Qrwin’s Fail-safe *N*, and Kendall’s τ. A symmetrical funnel plot, large Qrwin’s Fail-safe *N* (>5*k* + 10), and non-significant Kendall’s τ would indicate the absence of publication bias.

## Results

After applying the above criteria, 42 independent studies (9 for EI-burnout; 18 for SE-burnout; and 15 for EI-SE) from 40 manuscripts covering 5,665 teachers were retained and analyzed (please refer to the Supplementary Appendix for articles included). Apart from one study (Akbari and Tavassoli, 2011), which measured all of the three constructs (SE, EI, and burnout), all the studies measured either two of them. Out of the 42 studies, 39 were published manuscripts and 3 were dissertation projects. All of them were cross-sectional studies. Most of the studies were conducted in non-English speaking countries such as Iran and Turkey. They were published/completed between 2009 and the present. Participants were dominantly English as a Foreign Language (EFL) teachers [except for Kostić-Bobanović (2020), wherein teachers instruct a wide range of foreign languages] from public or private institutes and schools with 1–35 years of teaching experience. The most frequently used measures of EI, burnout, and SE were the Emotional Quotient Inventory (EQ-i; Bar-On, 2000), Maslach Burnout Inventory-Educators’ Survey (Maslach et al., 1996b), and Teachers’ Self-efficacy Scale (TSES; Tschannen-Moran and Hoy, 2001), respectively. Table 1 summarized the characteristics and effect sizes of included studies.

### Effect Size Analyses

The three sets of weighted average unattenuated correlations were all significant and in expected directions (see Table 2 for a summary). Under the random-effects model, the mean correlation between EI and burnout was −0.453, with a 95% CI from −0.587 to −0.294. The mean correlation between SE and burnout was −0.331, with a 95% CI from −0.456 to −0.193. Lastly, the mean correlation between SE and EI was 0.534, with a 95% *CI* from 0.440 to 0.617. The heterogeneity test showed *Q* was also significant indicating that the variance of effect sizes was greater than that originating from sampling error. *I*^2^ were all larger than 75%, indicating a large variance across study findings, which further necessitated the following moderation analyses.

**TABLE 2:** Summary of weighted effect sizes.

					Hetereogeneity			Publication bias	
		
Effect	*k*	N	Weighted mean effect size	95% CI	*Q*	*I* ^2^	*t* ^2^	Classic fail safe *N*	Kendall’s *t*
EI-burnout	9	1,021	−0.453***	[−0.587, −0.294]	65.445***	87.776	0.068	421	−0.472
SE-burnout	18	3,086	−0.331***	[−0.456, −0.193]	276.367***	93.849	0.094	2,007	0.170
EI-SE	15	2,086	0.534***	[0.440, 0.617]	107.062***	86.923	0.050	2,471	0.133

***p < 0.001.

### Publication Bias

For the three sets of effect sizes, our data suggested the non-existence of publication bias (see Table 2 for the indices). Funnel plots were roughly symmetrical indicating evenly distributed effect sizes around the mean. Fail-safe numbers were much larger than the criteria ranging from 421 to 2,471. Kendall’s τ ranged from −0.47 to 0.17 (*p* > *0.05*). Subgroup analysis on publication status regarding EI-burnout and EI-SE suggested that effect sizes of dissertations did not differ from those of published studies. Since all the studies on SE-burnout were published, publication status was not analyzed as a moderator in this case.

### Moderation Analyses

Moderator analyses were carried out, based on mixed-effects models, to evaluate the effects of publication type, school type, teacher type, educational stage, publication year, and gender (percentage of female teachers). When the moderators were continuous variables, slopes, standard errors, 95% CI, and *Q* statistics were estimated. When the moderators were categorical, mean correlations, 95% CI, and *Q* statistics were estimated. All the results were displayed in Table 3.

**TABLE 3:** Summary of moderation analyses.

Categorical moderators	N effect sizes	*r*	95% CI	*Q*

EI-burnout (k = 9)
Publication status				3.97
Published	8	−0.48***	[−0.62, −0.30]	
Unpublished	1	−0.25**	[−0.39, −0.10]	
School type				0.09
Private	5	−0.43***	[0.63, −0.18]	
Public	4	−0.48***	[−0.66, −0.25]	
EI stream				3.37
Trait	7	−0.50***	[−0.66, −0.29]	
Ability	2	−0.29***	[−0.39, −0.18]	
Continuous moderators	β	SE	95% CI	*Q*
Publication year	0.001	0.008	[−0.016, 0.017]	0.003
% female	0.964	0.197	[0.579, 0.135]	24.06***

**SE-burnout (k = 18)**

School type				3.29
Private	4	−0.447***	[−0.58, −0.29]	
Public	6	−0.004	[−0.46, 0.45]	
Mixed	5	−0.380***	[−0.53, −0.21]	
Educational stage				9.08*
High school	5	0.02	[−0.52, 0.55]	
Institute	5	−0.49***	[−0.61, −0.34]	
University	1	−0.12	[−0.36, 0.13]	
Mixed	4	−0.31***	[−0.42, −0.18]	
Continuous moderators	β	SE	95% CI	*Q*
Publication year	0.008	0.006	[−0.003, 0.019]	2.03
% female	0.386***	0.114	[0.162, 0.609]	11.45***

**SE-EI (k = 15)**

Publication status				0.005
Published	13	0.54***	[0.43, 0.63]	
Unpublished	2	0.53***	[0.30, 0.70]	
Teacher type				0.123
Pre-service	2	0.49**	[0.13, 0.73]	
In-service	13	0.54***	[44, 0.63]	
School type				0.079
Private	3	0.51***	[0.21, 0.72]	
Public	11	0.54***	[0.44, 0.64]	
EI stream				2.83
Trait	12	0.59***	[0.52, 0.65]	
Ability	2	0.65***	[0.55, 0.72]	
Continuous moderators	β	SE	95% CI	*Q*
Publication year	0.014	0.006	[0.002, 0.025]	5.12*
% female	−1.13	0.174	[−1.475, −0.791]	42.20***

*p < 0.05; **p < 0.01; ***p < 0.001.

For the three sets of effects, publication type did not relate significantly to the outcomes, suggesting that published journal articles reported similar effects to unpublished theses. Similarly, the EI stream showed no influence on the effect size. Teacher type did not significantly influence the outcome (SE-EI link), even though the effect among in-service teachers (*r* = 0.54) was larger than pre-service teachers (*r* = 0.49). Publication year was significantly related to the correlation between SE and EI (β = 0.014, *p* < 0.05). This indicated that newer studies reported a larger effect than older ones. School type seemed to not influence the correlations. However, focusing on the relationship between SE and burnout, the educational stage turned out to be a significant moderator with the effects of school type varying greatly, implying that for public high school and university teachers, SE was not as associated with burnout as counterparts in other types and stages of schools.

The percentage of females is significantly related to the size of all the three sets of effects, though in opposite directions. The relation between EI-burnout and SE-burnout became stronger when there were more female participants (β = 0.964, *p* < 0.001 for EI-burnout; β = 0.386, *p* < 0.001 for SE-burnout), respectively. Nevertheless, the relation between EI and SE became weaker when there were more female participants (β = −1.13, *p* < 0.001).

## Discussion

The current study aimed to quantitatively review the relationship between EI, SE, and burnout among foreign language teachers. The three main effects yielded were all statistically significant, indicating a moderate relation between SE and burnout and a large relation between EI and burnout, as well as between EI and SE. According to the benchmark recommended by Cohen (2013), correlation coefficients over 0.30 should be considered medium and over 0.50 being large. Thus, our data showed that EI was correlated with SE with a large magnitude, while SE and EI were correlated with burnout with a medium magnitude. The overall results indicated that foreign language teachers with more confidence in their ability in complementing teaching activities and more perceived ability in dealing with emotions were less susceptible to feelings of burned-out characterized by emotional exhaustion, depersonalization, and lack of personal accomplishment. Meanwhile, emotionally intelligent teachers tended to feel more capable of managing language learning classrooms and students.

The overall contribution of EI and SE on burnout was as expected and theoretically meaningful. According to the Job Demand-Resource theory (Bakker and Demerouti, 2007, 2017), job demands such as work stress could lead to exhaustion, whereas job resources such as social support could instigate work engagement. Later, many studies embedded personal resources into this model and generated the bulk of the evidence. For example, self-efficacy, self-esteem, and optimism could not only influence employees’ perception of job resources but also mediate the relationship between job resources and emotional exhaustion among engineers (Xanthopoulou et al., 2007). Career-calling moderated the relationship between academic stress and burnout among doctors (Creed et al., 2014). This study provided further support for the integration of personal resources into the Job Demand-Resource theory and extended the theory to the sample of foreign language teachers.

Additionally, this finding was in line with the conservation of resources (COR) theory, whose basic tenet is that humans are motivated to protect their available resources and broaden new resources (Hobfoll, 2011). In the face of work stress, teachers who were more emotionally conscious and capable tended to develop more confidence in completing teaching activities, and these positive beliefs help them to withstand emotional exhaustion. There was evidence that EI as a potential personal resource was associated with increased positive emotions (Zeidner et al., 2012) and resilience (Brackett and Katulak, 2006), and social support at work (Ju et al., 2015). In addition, teachers with high EI tended to have more control over teaching activities, to be more skillful in coping with stress, and appraise stress as a challenge rather than a threat (Mérida-López and Extremera, 2017). Thus, it could be tentatively concluded that teachers with high EI could extend more personal and work resources, which together protect themselves from being exhausted at work. In a similar vein, the protective role of SE on burnout was also not hard to understand. Compared with those with high SE, teachers with high SE were less likely to ruminate over their deficiencies, magnify potential threats (Bandura et al., 1999), have a conflict with school administrators and students, or elicit defensive behaviors that heighten burnout syndrome (Skaalvik and Skaalvik, 2007).

Empirically, the moderate correlation between EI and burnout also echoed previous reviews (Mérida-López and Extremera, 2017), but extended the literature by providing quantitative mean scores. The protective role of SE on burnout was consistent with previous meta-analyses reporting the negative relation between classroom management SE (one specific dimension of teachers’ self-efficacy) and burnout (Aloe et al., 2014). However, Aloe and colleagues’ studies focused on the relation between SE and the three dimensions of burnout rather than overall burnout. It would be interesting, when sufficient studies have accumulated, to revisit the components of these two constructs among foreign language teachers to determine specific contributions.

The significant averaged positive relationship between EI and SE was also noteworthy. Though not many studies focused on these two positive individual differences at the same time, literature documented explanations for such a relationship. EI was deemed as a predictor of SE since teachers who are more capable of identifying students’ emotions and facilitating students’ positive emotions may be more efficacious in their abilities to manage the classroom, and motivate students who feel bored or anxious in language learning (Moafian and Ghanizadeh, 2009). It would be interesting to investigate, however, which components of EI (e.g., intrapersonal or interpersonal; emotional identification or management) have the strongest link with specific SE beliefs.

The interrelationship between EI, SE, and burnout was robust because they held across a range of moderators including teacher type and school characteristics. Previous studies also reported the invariance between teacher EI and SE among teachers of different ages or teaching experiences (Rastegar and Memarpour, 2009). Having said that, gender effects are noteworthy. It seems that women language teachers could benefit more from perceived SE and EI in withstanding work-related psychological syndromes than men teachers. Also, emotional understanding and regulation ability could empower women teachers with more positive self-evaluation than men teachers. However, future studies are needed to further explore the reasons for the gender effect before meaningful interpretations could be provided.

### Implications

The current study advanced the literature in three ways: (1) It meta-analyzed the correlation between two positive psychological constructs (SE and EI) and burnout among foreign language teachers for the first time; (2) It served as the first meta-analysis synthesizing the correlation between SE and EI among language teachers; (3) It preliminarily explored potential moderators influencing the three sets of effects. The findings were statistically and practically significant. Imparting knowledge about teachers’ self-efficacy beliefs and empowering them with emotion management competence would be a promising direction for future teacher education and intervention. To combat burnout, it would be necessary to screen teachers’ SE and EI in the first step, develop courses and programs to facilitate teachers appropriately handling their emotions, evaluating their teaching competence, and, if possible, incorporate EI and SE as parts of teacher development. These practices should be specially targeted at women teachers.

Evidence showed that emotional literacy is especially important for teachers in the field of sentimental emotion-provoking social sciences as opposed to rational natural sciences (MacCann et al., 2020). Following the same logic, emotional intelligence is especially relevant for language teachers since they are expected to initiate and maintain classroom interaction by practicing the target language and, thus, in face of more emotional challenges. Turning to the value of SE, for foreign language teachers as non-native speakers, confidence in successful instruction is important because they confront dual sources of stress: the stress of uttering accurate sentences in the target language and the stress of delivering professional and in-depth knowledge on a certain topic. Without enough dose of such confidence, they are more susceptible to emotional drain at work.

### Limitations and Future Directions

There are some limitations to this study. Firstly, the effect sizes are cross-sectional. In other words, we do not know the causal inference regarding the direction of the relationships. The three constructs may contribute to each other reciprocally. Although previous studies suggested the conceptual influence of SE and EI on burnout, it is necessary to explore the directions in longitudinal studies. The mechanisms accounting for the effects also necessitate longitudinal studies. For example, it is reasonable to speculate that teacher burnout may affect their SE beliefs since SE was heavily hinged on previous experiences. Emotional exhaustion may reduce teachers’ confidence in teaching performance (Skaalvik and Skaalvik, 2007).

Secondly, we did not explore other important moderators due to a lack of relevant information. The relationship between EI, SE, and burnout may hinge on moderators such as teachers’ language proficiency, average age, years of teaching experience, and quality and quantity of language exposure, as well as countries, but such information is lacking in most studies included, which made it impossible to analyze their influences. We encourage researchers to report and account for these demographic/background variables to optimize teacher education and capture a more conclusive picture.

Finally, the effects reported in this study were correlations between general constructs. In other words, we did not examine a specific type of constructs (e.g., classroom management self-efficacy or emotion regulation ability) or dimension of constructs (e.g., emotional exhaustion). Components of EI may link with burnout and self-efficacy differently, but due to lack of enough information, we cannot tease out the nuance. Previous studies were not conclusive as to which components were more strongly correlated. Some studies found that teacher efficacy was more strongly related to reduced personal accomplishment (Aloe et al., 2014) whilst others found that it was more strongly related to depersonalization (Brown, 2012). Additionally, since self-efficacy is context- and domain-specific, teachers may be not equally efficacious in teaching functioning (Chan, 2008). Presumably, teachers’ general self-efficacy and teaching-related self-efficacy would relate to EI and burnout in a different vein. Therefore, it would be necessary to provide a more in-depth insight by examining correlations between overall scores but also specific subscales as well.

## Conclusion

To respond to the recent call for language teachers’ research in addition to that of language learners, the meta-analytic review synthesized findings on the interrelationship between language teachers’ EI, SE, and burnout. Results showed that the three constructs demonstrated moderate to large weighted average correlations which remained unchanged across a range of moderators. Teachers’ emotional state deserves further attention since it is transmittable and of great relevance in language learning classrooms. It would be beneficial if future pre-service teacher education and support at the workplace could target enhancing teachers’ EI and SE.

## Data Availability Statement

The original contributions presented in this study are included in the article/Supplementary Material, further inquiries can be directed to the corresponding author.

## Author Contributions

YBW drafted the manuscript. YYW provided research ideas. Both authors worked on the coding process and approved the submitted version.

## Conflict of Interest

The authors declare that the research was conducted in the absence of any commercial or financial relationships that could be construed as a potential conflict of interest.

## Publisher’s Note

All claims expressed in this article are solely those of the authors and do not necessarily represent those of their affiliated organizations, or those of the publisher, the editors and the reviewers. Any product that may be evaluated in this article, or claim that may be made by its manufacturer, is not guaranteed or endorsed by the publisher.
