# Supplementary material for: The Interrelationship Between Emotional Intelligence, Self-Efficacy, and Burnout Among Foreign Language Teachers: A Meta-Analytic Review
Source: Front Psychol. 2022 Jun 28;13:913638. doi: 10.3389/fpsyg.2022.913638 (PMC9274274; doi:10.3389/fpsyg.2022.913638)
Supplement: Supplementary file 1 [file Data_Sheet_1.docx]

**Appendix A** Studies included in the current meta-analysis

**# EI and burnout** (8 manuscripts; 9 independent samples)

1. Akabari, R., Tavassoli, K. (2011). Teacher efficacy, burnout, teaching style, and emotional intelligence: Possible relationships and differences. *Iranian Journal of Applied Linguistics*, 14(2), 31-61.

2. Alavinia, P., & Ahmadzadeh, T. (2012). Towards a reappraisal of the bonds between emotional intelligence and burnout. *English Language Teaching*, 5(4), 37-50.

3. Amirian, S. M. R., Masjedy, H., & Amirian, S. K. (2021). Reflections on English as a foreign language teacher burnout risk factors: The interplay of multiple variables. *Applied Research on English Language*, 10(1), 33-50.

4. Durhan, G. (2019). *The relationship between English teachers’ emotional intelligence and their burnout level*. Hacettepe University (master’s thesis).

5. Esmaili, R., Khojasteh, L., & Kafipour, R. (2018). The relationship between emotional intelligence and burnout among EFL teachers teaching at private institutions. *Social Sciences & Humanities*, 26(3), 1595-1616.

6. Heiran, A., & Navidinia, H. (2015). Private and public EFL teachers’ level of burnout and its relationship with their emotional intelligence: A comparative study. *International Journal of English Language & Translation Studies*, 3(3), 1-10.

7. Mahmoodi, M. H., & Ghaslani, R. (2014). Relationship among Iranian EFL teachers’ emotional intelligence, reflectivity and burnout. *Iranian Journal of Applied Language Studies*, 6(1), 90-116.

8. Vaezi, S., & Fallah, N. (2011). The relationship between emotional intelligence and burnout among Iranian EFL teachers. *Journal of Language Teaching and Research*, 2(5), 1122-1129.

**# SE and burnout** (17 manuscripts, 18 independent samples)

1. Akabari, R., Tavassoli, K. (2011). Teacher efficacy, burnout, teaching style, and emotional intelligence: Possible relationships and differences. *Iranian Journal of Applied Linguistics*, 14(2), 31-61.

2. Akhavanattar, F., & Ahmadi, S. D. (2017). The relationship of EFL teacher efficacy, job satisfaction, and work-related burnout. *Advanced Social Humanities and Management*, 4(1), 20-30.

3. Amirian, S. M. R., Masjedy, H., & Amirian, S. K. (2021). Reflections on English as a foreign language teacher burnout risk factors: The interplay of multiple variables.

4. Fathi, J. (2020). A structural model of teacher self-efficacy, resilience, and burnout among Iranian EFL teachers. *Iranian Journal of English for Academic Purposes*, 9(2), 14-26.

5. Ghasemzadeh, S., Nemati, M., & Fathi, J. (2019). Teacher self-efficacy and reflection as predictors of teacher burnout: An investigation of Iranian English language teachers. *Issues in Language Teaching*, 8(2), 25-50.

6. Ghorbanzadeh, A., & Rezaie, G. (2016). The relationship between English language teacher perfectionism, efficacy, and burnout. *International Journal of Foreign Language Teaching & Research*, 4(14), 97-106.

7. Gigasari, N. S., & Hassaskhah, J. (2017). The effect of social comparison tendencies on EFL teachers’ experience of burnout and instructional self-efficacy. *Cogent Psychology*, 4(1), 1-21.

8. Khani, R., & Mirzaee, A. (2014). How do self-efficacy, contextual variables and stressors affect teacher burnout in an EFL context? *Educational Psychology*, 35(1), 93-109.

9. Mardani, N., Baghelani, E., & Azizi, R. (2015). Exploring the relationship between self-efficacy and burnout: The case of Iranian EFL teachers. *Cumhuriyet Üniversitesi Fen Edebiyat Fakültesi Fen Bilimleri Dergisi*, 36(3), 3538-3548.

10. Mashhady, H., Fallah, N., & Gaskaree, B. L. (2012). The role of foreign language teachers’ self-efficacy in their burnout. *British Journal of Education, Society & Behavioral Science*, 2(4), 369-388.

11. Mede, E. (2009). An analysis of relations among personal variables, perceived self-efficacy and social support on burnout among Turkish EFL teachers. *Inonu University Journal of the Faculty of Education*, 10(2), 39-52.

12. Motallebzadeh, K., Ashraf, H., & Yazdi, M. T. (2014). On the relationship between Iranian EFL teachers’ burnout and self-efficacy. *Procedia-Social and Behavioral Sciences*, 98, 1255-1262.

13. Ozkara, B. (2019). An investigation into the relationship between Turkish EFL teachers’ self-efficacy and burnout level. *Journal of Family, Counseling, and Education*, 4(1), 12-24.

14. Roohani, A., & Iravani, M. (2020). The relationship between burnout and self-efficacy among Iranian male and female EFL teachers. *Journal of Language & Education*, 6(1), 173-188.

15. Safari, I. (2021). Relationship between Iranian EFL teachers’ self-efficacy and their burnout level in universities and schools. *International Journal of Foreign Language Teaching & Research*, 9(35), 25-38.

16. Topuzov, O. M., Malykhin, O. V., Aristova, N. O., & Shamne, A. V. (2020). Optimization of university department management: The increase of foreign language teachers’ self-efficacy by preventing job burnout. *New Educational Review*, 59, 59-71.

17. Yazdi, M. T., Motallebzadeh, K., & Ashraf, H. (2014). The role of teachers’ self-efficacy as a predictor of Iranian EFL teacher’s burnout. *Journal of Language Teaching and Research*, 5(5), 1198-1204.

**# SE and EI** (15 manuscripts, 15 independent samples)

1. Akabari, R., Tavassoli, K. (2011). Teacher efficacy, burnout, teaching style, and emotional intelligence: Possible relationships and differences. *Iranian Journal of Applied Linguistics*, 14(2), 31-61.

2. Amirian, S. M. R., Masjedy, H., & Amirian, S. K. (2021). Reflections on English as a foreign language teacher burnout risk factors: The interplay of multiple variables.

3. Amirian, S. M. R. & Behshad, A. (2016). Emotional intelligence and self-efficacy of Iranian teachers: A research study on university degree and teaching experience. *Journal of Language Teaching and Research*, 7(3), 548-558.

4. Karakas, M. (2016). *An examination of pre-service ELT teachers’ sense of self-efficacy, emotional intelligence and teacher knowledge as constituents of teacher identity construction*. Çanakkale Onsekiz Mart University (doctoral dissertation).

5. Koçoğlu, Z. (2014). Emotional intelligence and teacher efficacy: A study of Turkish EFL pre-service teachers. *Teacher Development*, 15(4), 471-484.

6. Kostic-Bobanovi, M. (2020). Perceived emotional intelligence and self-efficacy among novice and experienced foreign language teachers. *Economic Research-Ekonomska Istrazivanja*, 33(1), 1200-1213.

7. Mashhady, H. (2013). Toward an analysis of the bond between emotional intelligence and self-efficacy among EFL teachers. *Journal of English Language Teaching and Learning*, 5(11), 107-125.

8. Moafian, F., & Ghanizadeh, A. (2009). The relationship between Iranian EFL teachers’ emotional intelligence and their self-efficacy in language institutes. *System*, 37(4), 708-718.

9. Nikoopour, J., Farsani, M. A., Tajbakhsh, M., & Kiyaie, S. H. S. (2012). The relationship between trait emotional intelligence and self-efficacy among Iranian EFL teachers. *Journal of Language Teaching and Research*, 3(6), 1165-1174.

10. Özel, K. (2019). *The relationship between emotional intelligence, social intelligence and self-efficacy among preservice English teachers*. Hacettepe Univesity (master’s thesis).

11. Rastegar, M., & Memarpour, S. (2009). The relationship between emotional intelligence and self-efficacy among Iranian EFL teachers. *System*, 37, 700-707.

12. Moghadam, H. R. H. (2015). An investigation into the relationship between Iranian high school EFL teachers’ emotional intelligence and their self-efficacy. *International Journal of Social Science & Education*, 5(3), 509-518.

13. Sarkhosh, M., & Rezaee, A. A. (2013). How does university teachers’ emotional intelligence relate to their self-efficacy beliefs? *Porta Linguarum Revista Interuniversitaria de Didáctica de las Lenguas Extranjeras*, 21, 85-100.

14. Siyamaknia, N., Tabrizi, A. R. N., & Zoghi, M. (2013). On the relationship between emotional intelligence and teachers’ self-efficacy in high school and university contexts. *ELT Voices-India*, 3(6), 71-79.

15. Wossenie, G. (2014). A study on second cycle public primary school EFL teachers in Bahir Dar Town, Ethiopia. *International Journal of English and Literature*, 5(7), 155-164.

**Appendix B** PRISMA flow diagram of the searching process

Literature Search

Database

(PsycINFO, EBSCO, Eric, etc)

Reference lists of

reviews & included articles

Reference lists of

relevant reviews

Total number of records

identified (n=217)

Records (duplicates excluded)

screened (n=183)

identified (n=2337)

Full-text articles assessed for

eligibility (n=88)

Total excluded (n=95)

Reviews (n=8)

Not relevant (n=45)

Constructs not continuous (n=10)

Qualitative (n=22)

Full-text unavailable (n=5)

Not in English (n=5)

Total excluded (n=46)

Not enough information

to calculate ES (n=38)

Table and text conflictual (n=8)

Studies included

(n=3 dissertations; n=39 journal articles)

Reference lists of

relevant reviews
